# Cellular mRNAs access second ORFs using a novel amino acid sequence-dependent coupled translation termination–reinitiation mechanism

**DOI:** 10.1261/rna.041574.113

**Published:** 2014-03

**Authors:** Phillip S. Gould, Nigel P. Dyer, Wayne Croft, Sascha Ott, Andrew J. Easton

**Affiliations:** 1 School of Life Sciences, University of Warwick, Coventry CV4 7AL, United Kingdom; 2 Warwick Systems Biology Centre, University of Warwick, Coventry CV4 7AL, United Kingdom

**Keywords:** coupled translation, translation initiation, second ORF

## Abstract

Polycistronic transcripts are considered rare in the human genome. Initiation of translation of internal ORFs of eukaryotic genes has been shown to use either leaky scanning or highly structured IRES regions to access initiation codons. Studies on mammalian viruses identified a mechanism of coupled translation termination–reinitiation that allows translation of an additional ORF. Here, the ribosome terminating translation of ORF-1 translocates upstream to reinitiate translation of ORF-2. We have devised an algorithm to identify mRNAs in the human transcriptome in which the major ORF-1 overlaps a second ORF capable of encoding a product of at least 50 aa in length. This identified 4368 transcripts representing 2214 genes. We investigated 24 transcripts, 22 of which were shown to express a protein from ORF-2 highlighting that 3′ UTRs contain protein-coding potential more frequently than previously suspected. Five transcripts accessed ORF-2 using a process of coupled translation termination–reinitiation. Analysis of one transcript, encoding the CASQ2 protein, showed that the mechanism by which the coupling process of the cellular mRNAs was achieved was novel. This process was not directed by the mRNA sequence but required an aspartate-rich repeat region at the carboxyl terminus of the terminating ORF-1 protein. Introduction of wobble mutations for the aspartate codon had no effect, whereas replacing aspartate for glutamate repeats eliminated translational coupling. This is the first description of a coordinated expression of two proteins from cellular mRNAs using a coupled translation termination–reinitiation process and is the first example of such a process being determined at the amino acid level.

## Abstract (precis)

Polycistronic transcripts are rare in the human genome as unusual mechanisms are needed to translate the downstream ORFs, including leaky scanning, IRESs, or coupled termination–reinitiation mechanisms. Here the authors have devised an algorithm to identify mRNAs in the human transcriptome with two overlapping ORFs where a coupled termination–reinitiation mechanism might be relevant. Of the thousands of such transcripts identified, 22 of 24 were seen to express a protein from the second ORF suggesting that 3′ UTRs themselves have considerable coding potential. Five of these transcripts appeared to depend on a termination–reinitiation mechanism, and one of these depended on a specific aspartate-rich repeat peptide sequence at the carboxyl terminus of ORF1 for the coupling mechanism to be effective.

## INTRODUCTION

The eukaryotic translation machinery exploits a number of processes to control gene expression in a wide range of fundamental cellular processes (Aitken and Lorsch 2012). The majority of eukaryotic mRNAs are monocistronic, expressing a single polypeptide from the 5′ proximal open reading frame (ORF). If the sequence surrounding the first AUG is not favorable the ribosome may use leaky scanning to initiate translation at the next AUG to generate additional proteins (Kozak 1997). Ribosomes can be detected translating alternative reading frames (Wilson and Masel 2011; Michel et al. 2012). Additional translational initiation mechanisms include the use of internal ribosome entry sites (IRES), ribosomal shunting, and coupled translation (Ahmadian et al. 2000; Rogers et al. 2004; Spriggs et al. 2008). These mechanisms have the potential to expand the coding capacity of the genome.

In bacteria, where polycistronic mRNAs are common, ribosomes can scan bidirectionally around termination codons prior to reinitiation on upstream or downstream AUG codons (Adhin and van Duin 1990). In eukaryotes, termination of translation of a large 5′ proximal ORF followed by reinitiation of translation on the same mRNA to access a second ORF has been considered a rare event, first demonstrated with the hepatitis B virus P mRNA and also with artificially made mRNAs (Peabody and Berg 1986; Kozak 1989). In the other cases where reinitiation has been seen the upstream ORF frequently does not encode a substantive protein with a defined function and its presence generally reduces translation of the downstream ORF (Pöyry et al. 2004; Jackson et al. 2010). Examples have also emerged where the small upstream ORF has a biological role (Tautz 2009). The M2 mRNAs of all pneumoviruses contain two open reading frames, conserved in location though not in sequence. ORF-1 utilizes 60%–75% of the coding capacity of the mRNA and encodes the M2-1 protein product, the virus transcriptional activator (Collins and Wertz 1985; Ling et al. 1992; Ahmadian et al. 1999; Fearns and Collins 1999). We have demonstrated that ribosomes access and translate the second ORF in vivo in a controlled process by utilizing the three AUG codons located upstream of the ORF-1 termination codon, and expression from these initiation codons requires the prior termination of M2 ORF-1 translation (Ahmadian et al. 2000; Gould and Easton 2005). The RSV M2-2 protein produced by coupled translation is thought to be involved in control of the switch between virus RNA transcription and replication (Bermingham and Collins 1999). Extending the distance between the M2 ORF-1 termination codon and the M2 ORF-2 initiation codon from the 32-nt maximum observed in vivo to the 72-nt ablated translation of ORF-2. These data also demonstrated that translation of the second ORF is not due to the presence of an IRES sequence. The region of overlap of the M2 ORF-1 and ORF-2 alone is not sufficient to achieve coupled expression but also requires additional sequences located within the M2-1 ORF (Gould and Easton 2005). Importantly, in all of these studies the data were generated in cells in which no other virus genes were being expressed, indicating that the coupled translation process is, in principle, an option available to all cells. Coupled translation mechanisms have also been described in other pneumoviruses and in a number of caliciviruses and influenza B virus (Luttermann and Meyers 2007, 2009; Powell et al. 2008, 2011). The mechanism directing the coupling process differs in caliciviruses and influenza B. Here both require a short section of the mRNA, including a motif that binds to 18S rRNA, just upstream of the overlap (Luttermann and Meyers 2007, 2009; Powell et al. 2008, 2011).

Since the coupled translation process functions in the absence of any viral proteins we considered the possibility that coupled translation may occur with cellular mRNAs. We have screened the human genome for mRNAs containing overlapping ORFs where the second ORF was at least 150 nt in length and contained at least one AUG codon upstream of the ORF-1 stop codon, as seen with the pneumovirus M2 mRNAs. The algorithm would also identify candidate mRNAs that could utilize the mechanism seen in caliciviruses. This identified 4368 transcripts representing 2214 genes. We have demonstrated that the majority of the transcripts analyzed (22 of 24 tested) express proteins from ORF-2 and that five of these genes achieve this using a coupled translation process previously described only in viral transcripts.

The mechanism identified here for the five human transcripts was different from the two previously characterized viral mechanisms. Here, the amino acid sequence at the carboxyl terminus of the protein encoded by ORF1 modulates the coupling processes.

## RESULTS

### Identification of additional coding capacity within cellular transcripts

**FIGURE 1.**
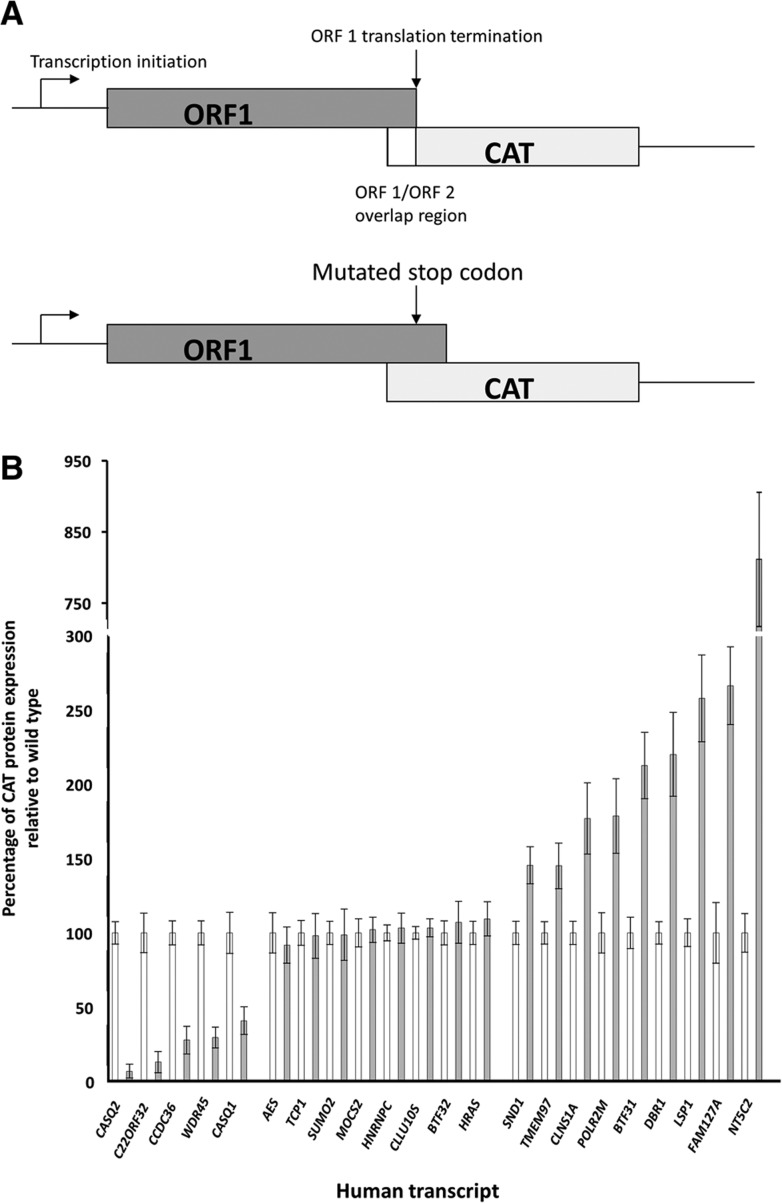
(*A*) Diagrammatic representation of the reporter gene construct used in the ORF-2 expression assays (not to scale). The ORF-2 coding region was replaced with the coding region of the chloramphenicol acetyl transferase (CAT gene). The ORF-1 sequence was unaltered. In the STOP mutants the termination codon of ORF-1 was mutated moving termination of ORF-1 downstream 36 nt. (*B*) Expression of the CAT protein from ORF-2 in constructs derived from the mRNA transcripts identified and listed in Supplemental Table 1. The expression from the wild-type gene construct is shown in the white columns and expression from the associated STOP mutant in which the ORF-1 translation termination codon was mutated are shown in the gray columns. The expression is given as a percentage of that seen with the nonmutated wild-type construct which was set as 100%. Error bars indicate the standard deviation of at least three independent experiments, with each performed in triplicate within an experiment.

We selected 24 human transcripts from a candidate list for analysis (full details supplied in Supplemental Table 1). The selection was based on the gene demonstrating a high probability of translational coupling using the scoring algorithm. The transcript encoding MOCS2 has previously been shown to access ORF-2 using leaky scanning to synthesize a component of the functional molybdopterin synthase enzyme and this was included as a control (Sloan et al. 1999; Stallmeyer et al. 1999). All other second ORFs were previously uncharacterized and no protein products from these ORFs have been described. Expression from ORF-2 was investigated by insertion of a CAT reporter gene lacking its endogenous AUG initiation codon and detection using an ELISA as described previously (Fig. 1A; Ahmadian et al. 2000; Gould and Easton 2005, 2007). This showed that 22 of the initial 24 transcripts studied expressed the CAT protein product while two transcripts, *MYADM* and *ARSD*, did not (Supplemental Table 1). Thus 92% of transcripts screened were able to express a protein from the additional ORF.

### Translational regulation of ORF-2

To screen for the utilization of coupled translation to access the ORF-2 the first nucleotide of the stop codon of ORF-1 in each construct was mutated. The next in-frame stop codon was 36 nt further downstream within the CAT ORF, resulting in a larger ORF-1 product (Fig. 1A). These were called STOP mutants. In previously characterized mRNAs where coupled translation termination–reinitiation occurs the expression level from ORF-2 is severely reduced when the ORF-1 stop codon is mutated as the ribosome must translocate upstream following termination of translation of ORF-1 and this is a distance-dependent phenomenon (Ahmadian et al. 2000; Luttermann and Meyers 2007). The effect of the STOP mutation also demonstrates that neither ribosomal scanning nor an IRES are responsible for the translation of ORF-2 as neither process would be affected by a mutation downstream from the translation initiation codon of ORF-2. Similarly, the process cannot be due to translation of degraded mRNA as this would not be affected by the STOP mutations. The average level of CAT protein expression of the appropriate wild-type control wells was set at 100% and the average level of expression for the relevant STOP mutants was compared with this. Analysis of the data from the STOP mutants identified five mRNAs in which the mutant showed a significant reduction in expression from ORF-2 to 10% to 41% of the original high levels demonstrating that coupled translation occurs in these cases. This is consistent with previous observations with the RSV M2 mRNA in which the level of reduction depends on the distance between the ORF-1 stop codon and the 5′ proximal ORF-2 initiation codon (Ahmadian et al. 2000). The remaining genes for which ORF-2 expression was seen fell into two distinct categories. In eight genes the introduction of a point mutation to move the ORF-1 stop codon further downstream generated no significant difference in the level of expression from ORF-2 (Fig. 1B). For these genes the ORF-2 may be accessed by known translation initiation mechanisms such as ribosome scanning or internal ribosome entry but this was not investigated further. A third group of nine genes was identified in which the mutation of the ORF-1 stop codon resulted in a significant increase in expression from ORF-2 (Fig. 1B). The most dramatic example was the *NT5C2* transcript. While the level of CAT protein produced from the wild-type *NT5C2* construct was very low (260 pg per 10^6^ cells) the STOP mutant produced approximately ninefold higher levels (2400 pg per 10^6^ cells). It should be noted that expression levels from ORF-2 differed for the various mRNAs with those for the *AES*, *TCP1*, *BTF31*, *FAM127A*, and NT5C2 genes being the lowest (Supplemental Table 1).

### Coupled translation termination–reinitiation in cellular genes requires an aspartate-rich motif in the ORF-1 protein

**FIGURE 2.**
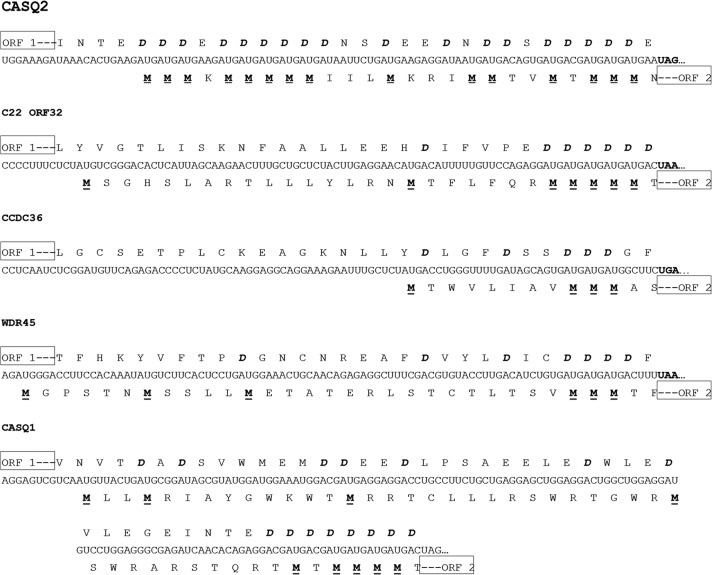
The sequences and coding capacity of the regions of overlap between ORF-1 and ORF-2 of the five genes shown to access ORF-2 using a coupled translation termination–reinitiation process. The multiple aspartate residues encoded by ORF-1 are shown in bold and italics and the potential AUG initiation codons for ORF-2 are shown in bold and underlined.

None of the mRNAs that use coupled translation termination–reinitiation to access ORF-2 contained a sequence upstream of the second ORF that was homologous to the sequences of the virus mRNAs shown to use coupled translation. Comparison of the sequences within and adjacent to the region of overlap between ORF-1 and ORF-2 of these five genes is shown in Figure 2. In all five genes the ORF-2 is in the +1 reading frame with respect to ORF-1. The second ORFs of these genes have several potential initiation methionine codons as this was one of the parameters used to rank the candidates identified by the basic algorithm. However, a striking aspect of the sequences of all five mRNAs is that the AUG codons are present in sequential runs of three or more. The most dramatic example of this is seen with the *CASQ2* gene where there are a total of 15 potential initiation codons in close proximity with three areas each containing three or more AUG codons. This is similar to the situation with the RSV M2 mRNA where there are three codons at the beginning of the ORF-2 and all have been shown to be utilized (Ahmadian et al. 2000). Subsequent analysis described below indicated that coupled translation in the *CASQ2* mRNA did not require all of the ORF-2 AUG codons. A consequence of the sequences in these overlap regions that can be seen in Figure 2 is that the carboxyl terminus of the proteins encoded by the ORF-1 contain a large number of aspartic acid residues present in short runs.

**FIGURE 3.**
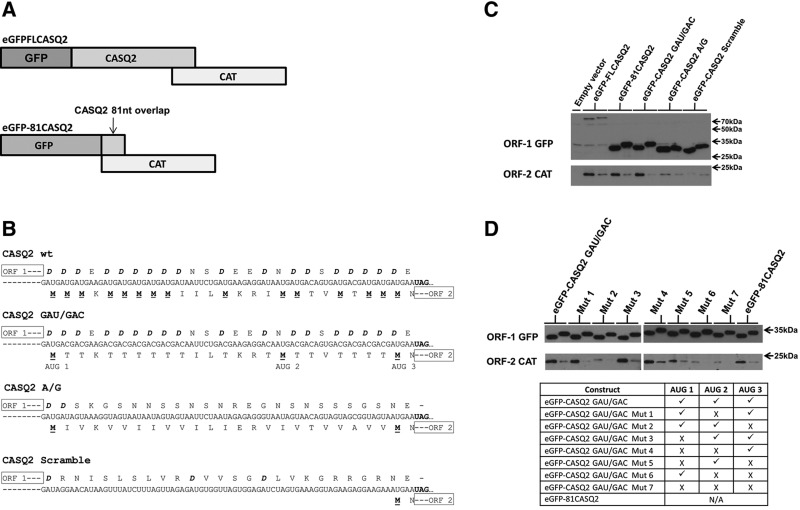
(*A*) Diagrammatic representation of the constructs used to investigate the effect of mutations on the expression of ORF-2. In all constructs the coding region of ORF-2 was replaced with that of the CAT gene. See text for details. (*B*) The sequences of the calsequestrin 2 (*CASQ2*) 81-nt sequence from the region of overlap between ORF-1 and ORF-2 subjected to mutation. The multiple aspartate residues of ORF-1 are shown in bold and italics and the potential initiation methionine residues of ORF-2 are shown in bold and underlined. The wild-type unaltered sequence is shown (CASQ2 wt) together with the CASQ2 GAU/GAC mutant that retains the multiple aspartate residues but lacks the multiple ORF-2 initiation codons, the CASQ2 A/G mutant that lacks the multiple ORF-1 aspartate and ORF-2 methionine residues, and the CASQ2 Scramble mutant that retains the nucleotides from the overlap region that have been randomly distributed to remove the multiple aspartate residues from ORF-1 and the multiple methionine residues from ORF-2. These mutants replaced the 81-nt *CASQ2* overlap shown in *A*. (*C*) Detection of GFP and CAT protein in lysates of cells transfected with expression plasmids by Western blot. GFP-specific and CAT-specific monoclonal antibodies were used to detect protein expression. The protein encoded by the eGFP-FLCASQ2 construct and detected by the anti-GFP antibody is 73.5 kDa because it is a fusion product of eGFP and CASQ2. Cells were transfected with the plasmid construct indicated. Lanes on the *left* of each pair are the constructs identified and lanes on the *right* of each pair are the appropriate STOP mutant. (*D*) Detection of GFP and CAT protein in cells transfected with expression plasmids as in *C*. Cells were transfected with the eGFP-CASQ2 GAU/GAC plasmid construct shown in *B* and with mutants derived from it in which one or more of the three AUG codons of ORF-2 labeled 1, 2, and 3 in the eGFP-CASQ2 GAU/GAC construct in *B* were mutated to ACG, AGC, and AGA, respectively. Lanes on the *left* of each pair are the parental construct and lanes on the *right* of each pair are the appropriate STOP mutant. The table *below* summarizes the presence (✓) and/or absence (X) of each of the three AUG codons in the mutants.

The translational coupling mechanism was investigated further using the *CASQ2* gene as a representative. As a first step we generated a construct in which the sequence for eGFP was fused at the 5′ end in frame with ORF-1 to generate an amino terminal extension of the CASQ2 protein shown diagrammatically in Figure 3A. The ORF-2 was replaced with the CAT gene as before. In this construct (eGFP-FLCASQ2 wt) the modified ORF-1 was 723 nt longer than the wild-type CASQ2 ORF. Thus, any potential for leaky scanning would be considerably reduced. A STOP mutant was generated for this construct (eGFP-FLCASQ2/STOP) as described above. Following transfection the expression of eGFP-CASQ2 fusion protein from ORF-1 and CAT protein from ORF-2 were detected directly by Western blotting using monoclonal antibodies. Figure 3C shows that the eGFP-CASQ2 fusion protein of 73.5 kDa from eGFP-FLCASQ2 wt was detected (ORF-1GFP). Mutation of the stop codon increased the size of eGFP by1.7 kDa as expected. The CAT protein expression from ORF-2 in the wild-type construct was dramatically reduced in the eGFP-FLCASQ2/STOP mutant, indicating that ORF-2 was accessed by coupled translation termination–reinitiation (Fig. 3C, ORF-2 CAT). To determine whether the sequences within the *CASQ2* gene are required for the coupling process a further construct was generated in which the region of the CASQ2 ORF-1 up to the position of the first ORF-2 AUG codon was entirely replaced with the gene encoding eGFP and the ORF-2 sequence was replaced with the CAT reporter gene. This left only the 81-nt CASQ2 ORF-1/ORF-2 overlap region flanked by the two reporter genes (eGFP-81CASQ2 wt) (Fig. 3A). An eGFP-81CASQ2/STOP mutant was also generated. The eGFP and CAT proteins were detected by Western blot and the eGFP expressed from the STOP mutant was increased in size demonstrating that the mutation had extended ORF-1 (Fig. 3C). The eGFP-81CASQ2 fusion protein was more readily detected in comparison to the eGFP-FLCASQ2 fusions. This is likely the result of the folded full-length chimeric protein masking the epitope sites. CAT protein production was not affected indicating that the mRNA was not being degraded (Fig. 3C). Importantly, ORF-2 CAT expression was reduced significantly in the STOP codon mutant (Fig. 3C, ORF-2 CAT). The level of reduction in CAT expression seen when comparing transfection with eGFP-81CASQ2 and eGFP-81CASQ2/STOP was consistent with the data showing a 90% reduction for the construct containing the authentic CASQ2 ORF-1 (Fig. 1B). This confirms that the overlap region of the *CASQ2* ORF-1 and ORF-2 alone is sufficient to direct coupled translation termination–reinitiation. The sizes of the various GFP proteins also confirmed that the expression of the CAT protein did not arise as a result of a translational frame-shifting event in any of the constructs. It was possible to utilize the construct eGFP-81 CASQ2 to quantify the coupling efficiency of the CASQ2 overlap as purified eGFP and CAT protein were available. Over three experimental repeats we found that CAT protein levels were 82-fold lower than eGFP (Supplemental Fig. 1) which is consistent with that seen for pneumoviral M2 mRNAs although reinitiation on calicivirus subgenomic mRNAs is an order of magnitude more efficient at 10%–20%.

To further investigate the mechanism of coupled translation used by the overlap region of the *CASQ2* gene, specific mutations were introduced into the region of overlap between ORF-1 and ORF-2 in the construct expressing GFP from ORF-1 and CAT from ORF-2 (Fig. 3A,B). In the first mutant (eGFP-CASQ2 GAU/GAC) the sequence was altered to replace the GAU codon for aspartate with GAC so that the amino acids encoded by ORF-1 were unaltered but the multiple AUG methionine codons of ORF-2 were absent leaving the first, last, and one central methionine (Fig. 3B). A second mutant was constructed in which 27 A↔G nucleotide transitions were introduced to eliminate the runs of aspartate in the ORF-1 reading frame and to also remove the multiple methionine codons in the ORF-2 reading frame, leaving only the first and last (Mutant A/G) (Fig. 3B). In the final mutant the overlap region was randomly scrambled (CASQ2 Scramble) (Fig. 3B), retaining the same number and proportion of all four nucleotides present in the wild-type sequence. In the CASQ2 Scramble sequence ORF-1 terminated translation at the same position as in the wild-type *CASQ2* ORF-1 and left the ORF-2 AUG initiation codon closest to the ORF-1 termination point, but the overlap region lacked the runs of aspartate residues. For each of these constructs a STOP mutant was also produced and the eGFP protein increased in size as expected (Fig. 3C, ORF-1 GFP). Figure 3C (ORF-2 CAT) shows that coupled expression of CAT protein from ORF-2 in construct eGFP-CASQ2 GAU/GAC was significantly reduced by moving the termination codon of ORF-1 in the associated STOP mutant. This indicates that the coupling process was not dependent on the precise nucleotide sequence of the overlap region and that the presence of the multiple AUG initiation codons for ORF-2 were not essential for the coupling to occur. In the mutants where the overlap sequence was mutated to eliminate the aspartate residues in ORF-1 either by specifically mutating codons or by randomizing the sequence, translational coupling was eliminated as demonstrated by lack of change in the low level of CAT protein expressed from ORF-2 when the ORF-1 termination codon was moved downstream in the relevant STOP mutants (Fig. 3C, ORF-2 CAT).

A noticeable feature of the analysis of the CASQ2 mutant mRNAs is that the CAT protein produced from ORF-2 appears as a discrete product despite variation in the number of potential AUG initiation codons for ORF-2 that might be expected to generate a heterogeneous set of proteins. This suggests that the reinitiation event may preferentially use one initiation codon. To investigate this we carried out further mutational analysis on the CASQ2 GAU/GAC mutant that contains three AUG initiation codons. The initiation codons were each mutated singly or in all possible combinations, as summarized in Figure 3D, and matched STOP mutants in which the ORF-1 stop codon was also mutated were also generated. Western blot analysis showed that termination–reinitiation of ORF-2 occurred readily when either only AUG 2 (in Mut 5) or only AUG 3 (in Mut 4) was present (Fig. 3D). The CAT protein synthesized from AUG2 migrated marginally slower than that from AUG3 as expected from the predicted sizes of the proteins. When only AUG 1 was present (in Mut 6) the level of expression from ORF-2 was the same as seen in the absence of any AUG codons (Mut 7) (Fig. 3D). The intensity of the CAT protein product was consistently greatest with Mut 4, than Mut 5 and this, together with the absence of expression from Mut 6 strongly suggests that there is a preference for the most proximal AUG initiation codon to the ORF-1 translation termination site.

**FIGURE 4.**
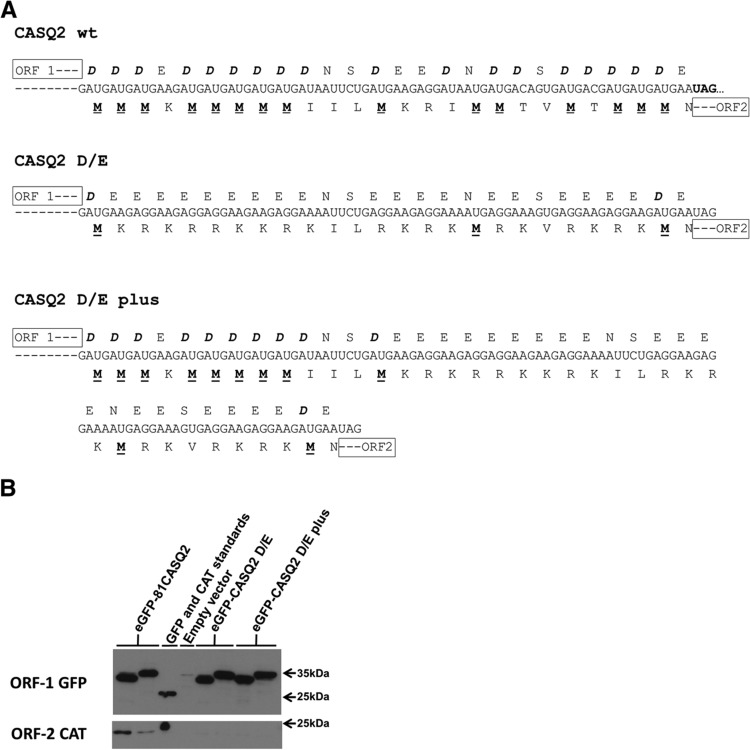
(*A*) The sequences of the calsequestrin 2 (*CASQ2*) 81-nt sequence from the region of overlap between ORF-1 and ORF-2 subjected to mutation. The multiple aspartate residues of ORF-1 are shown in bold and italics and the potential initiation methionine residues of ORF-2 are shown in bold and underlined. The wild-type unaltered sequence is shown (CASQ2 wt) together with the CASQ2 D/E mutant overlap in which the multiple aspartate residues were altered to glutamate residues and the CASQ2 D/E plus mutant in which nine aspartate residues at the amino terminal of the overlap region were maintained followed by the glutamic acid motif used in the CASQ2 D/E construct. (*B*) Detection of GFP and CAT protein in lysates of cells transfected with expression plasmids by Western blot. GFP-specific and CAT-specific monoclonal antibodies were used to detect protein expression. Cells were transfected with the plasmid construct indicated. Lanes on the *left* of each pair are the constructs identified and lanes on the *right* of each pair are the appropriate STOP mutant. Controls were GFP and CAT protein standards and lysate from cells transfected with empty expression vector, as indicated.

To unambiguously show that it was the aspartate-rich region that promotes coupling two final pairs of constructs were generated. As with other constructs the first and last aspartic acids that define the start and finish of the overlap region and provide start codons for ORF2 along the central AUG were maintained. The construct CASQ2 D/E replaced the aspartates with glutamates, thus producing a negatively charged homopolymer carboxyl terminus (Fig. 4A). An additional construct (CASQ2 D/E plus) maintained nine aspartate residues at the amino-terminal of the overlap region followed by the glutamic acid motif used in CASQ2 D/E. STOP mutants of both constructs were also generated to screen for coupled translation. The mutations had no effect on ORF1 (ORF-1 GFP) expression (Fig. 4B). Neither of the D/E construct pairs were able to express the second ORF protein (Fig. 4B). This suggests that the aspartic acid motif must be located at or near the carboxyl terminus of the ORF-1 protein within the overlap.

## DISCUSSION

The data presented here demonstrate for the first time that cellular genes express proteins from second ORFs in mRNAs using a coupled translation termination–reinitiation process. The process of coupled translation termination–reinitiation requires that a ribosome that completes translation of a 5′ proximal ORF translocates to an upstream AUG initiation codon to reinitiate translation of a second ORF. To date this process has been identified only in a limited number of virus-encoded mRNAs that use one of two possible mechanisms to achieve expression of the second ORF: In the RSV M2 mRNA the mechanism requires the presence of a highly structured region ∼150 nt upstream of the second ORF and in the caliciviruses the mechanism requires the presence of a short sequence homologous to the 18S rRNA ∼70 nt upstream of the second ORF. The coupled translation achieves two results, firstly it increases the coding capacity of the genome, and secondly the proportions of the two proteins produced from the mRNA and synthesized in stoichiometrically regulated amounts determined by the efficiency of the coupling process. Viruses use cellular translation machinery during infection and this raises the possibility that cellular genes may also use coupled translation to direct the synthesis of additional proteins.

The analysis described here investigated 24 mRNA transcripts representing 4368 candidate transcripts containing key features found in the viral mRNAs using coupled translation termination–reinitiation (Ahmadian et al. 2000; Gould and Easton 2005, 2007; Luttermann and Meyers 2007, 2009). Expression from second ORFs present in 22 of the 24 (92%) transcripts demonstrates that many more cellular transcripts access second ORFs to produce novel proteins than has previously been suggested. Expression levels from ORF-2 varied between the transcripts with a range of from 200 pg to 40 ng per 10^6^ cells (Supplemental Table 1). Several mechanisms have been described by which ribosomes can access second ORFs such as leaky scanning, ribosomal shunting, or by use of an upstream IRES and we identified a proportion (9/24) of the transcripts analyzed where the data were compatible with one or more of these processes (Fig. 1B). However, a further subset showed an unexpected profile in which there was a marked increase in ORF-2 expression when the ORF-1 stop codon was mutated (Fig. 1B). The reasons for this are not yet clear but it may be due to stalled termination of translation of ORF-1 inhibiting reinitiation. This may be similar to the situation described for the cytomegalovirus UL4 gene in which a delay in cleavage of the final aminoacyl tRNA peptidyl bond in a protein encoded by a short ORF upstream of the main ORF results in stalling of termination and subsequent reduction in translation of the primary product (Degnin et al. 1993; Janzen et al. 2002). In the situation of an overlapping ORF-1 and ORF-2 this may also lead to physical occlusion of the ORF-2 initiation codon to ribosomes that would generate the result seen.

In the pneumovirus M2 mRNA the two proteins produced by the single mRNA through the coupling process are functionally linked with one being a transcriptional activator and the other an inhibitor with the linked expression providing a level of previously unknown control of coordinated expression (Bermingham and Collins 1999; Fearns and Collins 1999). Further investigation of the cellular ORF-2 protein products may provide interesting insights into similar control processes. It is also possible that the second ORFs in these transcripts are involved in the process of de novo gene birth (Carvunis et al. 2012).

Five of the transcripts accessed ORF-2 by a process of coupled translation termination reinitiation (Fig. 1B). None of the genes shown to access ORF-2 by coupled translation had any sequence identity with the various virus mRNAs shown to use coupled translation termination–reinitiation (Kupfermann et al. 1996; Ahmadian et al. 2000; Gould and Easton 2005, 2007; Luttermann and Meyers 2007, 2009). Most strikingly, all five transcripts contained multiple GAUGAU repeats in the region of overlap between ORF-1 and ORF-2 with the AUGs forming the initiation codons of ORF-2 and the carboxyl terminus of the ORF-1 proteins containing multiple aspartic acid residues (Fig. 2). This, together with the observation that the 81-nt overlap region of the *CASQ2* mRNA alone is capable of directing the coupling process indicates that the cellular mRNAs use a novel mechanism to direct the coupling process (Fig. 3). In the CASQ2 mRNA the data in Figure 3D strongly suggest that the initiation of ORF-2 preferentially occurs at a specific, or limited number, of the available AUG initiation codons and while the 5′ proximal ORF-2 AUG codon can be used there is a preference for the codon(s) nearest to the ORF-1 stop codon. The process is therefore a length-dependent one, as is seen with the RSV M2 mRNA.

**FIGURE 5.**
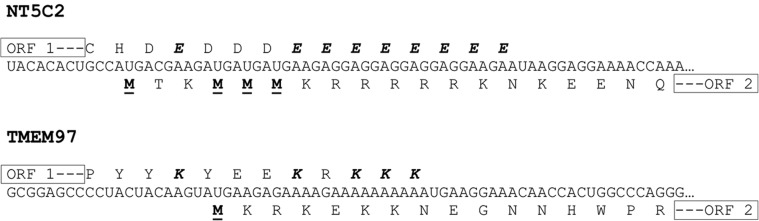
Sequences in the overlap region between ORFs 1 and 2 of the *NT5C2* and *TMEM97* genes. The glutamate and lysine-rich regions of the terminal regions of the ORF-1 proteins are highlighted in bold and italics. The potential initiation methionine residues of ORF-2 are shown in bold and underlined.

The presence of homopolymer runs of aspartate of the *CASQ2* transcript is essential for coupled translation to occur for this mRNA, further confirming that the process used is novel. The data presented in Figure 4 suggest that the aspartate motif must be located at or near the carboxyl terminus of the ORF-1 protein. The aspartate motif is located in this region in all of the mRNAs shown to direct coupled translation (Fig. 2). The presence of such extensive homopolymer runs of amino acids in proteins using the same codon is extremely unusual and is likely to have consequences for the translation of the mRNA. One possible consequence is that translation may be slowed if charged tRNAs cannot be provided rapidly. Also, the interaction between the nascent polypeptide and the ribosomal exit tunnel can directly affect translation, including causing stalling (Kramer et al. 2009). Stalling the ribosomes translating ORF-1 may be a necessary requirement to ensure that the terminating ribosomes are able to move in a 5′ direction before reinitiating translation at the start codon for ORF-2. The possibility that the presence of sequences rich in other single amino acids are sufficient to direct coupled translation was excluded, as replacement of the aspartate residues with multiple serine or glutamate residues eliminated the CASQ2 translational coupling (Figs. 3B,C, 4). Similarly, in the *NT5C2* and *TMEM97* genes (Fig. 1B) the overlapping regions between the ORF-1 and ORF-2 were rich in glutamate and lysine, respectively (Fig. 5), and while both expressed CAT protein from ORF-2, neither showed evidence of coupled translation. Taken together these data indicate that it is the presence of multiple aspartic acid residues in the carboxyl terminus of the ORF-1 protein and not the nucleotide sequence of the overlap region that is critical for coupled termination–reinitiation in the CASQ2 gene.

These data indicate that our understanding of the coding capacity of the human genome is not yet complete and that if the high proportion of mRNAs identified in this study utilizing second ORFs to produce protein products is representative of the several thousand genes identified as containing overlapping cellular ORFs, the scale of this is likely to be considerably higher than previously suspected. Many of these second ORF proteins will be produced by known processes such as leaky scanning, internal initiation with or without the use of IRES sequences, or ribosomal shunting. However, the data here suggest that coupled translation termination–reinitiation is also a significant translational control mechanism available to eukaryotic cells that will direct the synthesis of two proteins simultaneously in stoichiometrically regulated amounts. This suggests that the protein products are likely to be involved in related functions as seen with the RSV M2 proteins. The data also demonstrate that cells use at least two distinct mechanisms in which the overlap region between two ORFs with or without the need for additional upstream sequences can be utilized.

## MATERIALS AND METHODS

An algorithm was generated to search the complete list of alternative transcripts from the Ensembl release 55 of the human genome for transcripts where there were two ORFs in separate reading frames that were at least 150 nt in length that overlapped by between 4 and 120 nt. By definition the second ORF contained at least one AUG codon. If multiple start codons existed within the 120-nt overlap, then the start codon giving the smallest overlap was used to define the start of the second ORF. A BLAST search (release version 2.2.26) was performed of the protein sequences for each pair of ORFs against release 2012_3 of the UniProt/Swiss-Prot reference set of nonredundant protein sequences from multiple organisms. Transcripts where both ORFs matched a single known protein with E-values of <10^−5^, and transcripts where the first ORF did not match a known sequence with less than an E-value of 10^−5^ were rejected on the basis that there is likely to have been a misannotation or a misidentification of the first ORF. Transcripts were assigned a probability score based on the joint probability of a number of features that are believed to be associated with coupled translation based on the features of the RSV M2 mRNA. The less likely the feature, the greater the assumed likelihood of coupled translation. However, the algorithm would also identify candidate transcripts that utilize an alternative coupled translation termination–reinitiation mechanism such as that used by the caliciviruses. It was believed that the number of start codons, and the number of such codons followed by an A or C both influence the likelihood of coupled translation so that two of the factors contributing to the score were the probability of seeing at least the observed number of start codons in the 120 nt preceding the end of the second ORF and the probability of there being at least the observed number of trailing A and C residues associated with these start codons. It was assumed that longer second ORFs are an indication that the ORF corresponds to a biologically active protein, so a further factor is the probability of an ORF of that length or longer. Finally, it was assumed that the shorter overlaps are more likely to result in coupled translation so the final factor is the probability of an overlap length that is less than the one observed. These scores were used to order the candidate transcripts. The transcripts examined include some that were identified by early versions of the program but not by the current version as a result of updates to the reference sequences and the BLAST program. The current program identifies all of the transcripts where translational coupling has been verified experimentally as described below. The algorithm and software are available from the investigators.

The transcripts used in the study were those for calsequestrin 2 (cardiac muscle) (*CASQ2*: GenBank accession number NM_001232.3), chromosome 22 ORF 32 (*C22ORF32*: NM_033318.4), coiled-coil domain containing 36 (*CCDC36*: NM_001135197.1), WD repeat domain 45 (*WDR45:* NM_007075), calsequestrin 1 (*CASQ1:* NM_ 001231), amino-terminal enhancer of split (*AES;* NM_198969), t-complex 1 (*TCP1:* NM_030752), SMT3 suppressor of mif two 3 homolog 2 (*SUMO2*: NM_006937), molybdenum cofactor synthesis 2 (*MOCS2*: NM_176806), heterogeneous nuclear ribonucleoprotein C (C1/C2) (*HNRNPC*: NM_031314), chronic lymphocytic leukaemia up-regulated one opposite strand (*CLLU1OS*: NM_001025232), basic transcription factor 3 (*BTF32*: NM_001207), v-Ha-ras Harvey rat sarcoma viral oncogene homolog (*HRAS*: NM_176795), staphylococcal nuclease, and tudor domain containing 1 (*SND1*: NM_014390), Transmembrane protein 97 (*TMEM97*: NM_014573.2), chloride channel, nucleotide-sensitive, 1A (*CLNS1A*: NM_001293), RNA polymerase II polypeptide M (*POLR2M*: NR_027390.1), basic transcription factor 3 (*BTF31*: NM_001037637), debranching enzyme homolog 1 (*DBR1*: NM_016216), lymphocyte-specific protein 1 (*LSP1*: NM_002339), family with sequence similarity 127, member A (*FAM127A*: NM_001078171), 5′-nucleotidase, cytosolic II (*NT5C2*: NM_012229), myeloid-associated differentiation marker (*MYADM*: NM_001020818), arylsulfatase D (*ARSD*: NM_001669.2).

For cloning, PCR reagents were obtained from Promega and reactions were carried out using Pfu. Primers were obtained from IDT and are listed in Supplemental Tables 2 and 3. The cDNA clones of the genes of interest were obtained from commercial suppliers (Origene or Source Bioscience IMAGE clones) or synthesized from cDNA generated from HEp2 cell lines in our laboratory using standard protocols. The first ORF from each gene was amplified by PCR and the CAT reporter gene was inserted to replace the coding region of the second ORF in each gene immediately downstream from the overlap between ORF-1 and ORF-2. The entire construct was inserted into pBlueScribe as previously described (Ahmadian et al. 2000; Gould and Easton 2005). This general structure of the constructs is illustrated in Figure 1A including the stop mutant. For each construct an associated STOP mutant was generated by using an alternative reverse primer to that used to clone the wild type designed to mutate the ORF-1 translation termination codon, altering the sequence such that termination of ORF-1 at that point was eliminated and translation then terminated 36 nt further downstream within the CAT gene ORF. In total, 48 plasmid constructs were prepared and the sequences of all plasmids were verified prior to use.

The eGFP gene was obtained from plasmid peGFP (Clontech) and was cloned upstream and in-frame with either the full-length *CASQ2* ORF or the *CASQ2* 81-nt overlap. The CAT reporter was cloned downstream in frame with the ORF2 start codons generating plasmids eGFP-FLCASQ2 wt and eGFP81CASQ2, respectively (Fig. 3A). Mutations of the 81-nt *CASQ2* overlap region were generated using synthetic primers which when annealed in overlapping PCR created the novel sequence as shown in Figure 3B replacing the wild-type 81-nt *CASQ2* overlap sequence. Reciprocal STOP mutants were generated for all constructs.

Transfections of HEp2 cells were carried out in triplicate and repeated a minimum of three times and CAT protein detection by ELISA was carried out as described previously (Gould and Easton 2007). The antibodies used for direct detection of proteins in Western blots were for CAT #ab50151 and for GFP #ab290 (AbCAM). All lanes received the same amount of protein lysate. All Western blots were repeated in three independent experiments and a representative example is shown.

## SUPPLEMENTAL MATERIAL

Supplemental material is available for this article.
